# Nitric oxide/cGMP pathway signaling actively down-regulates α_4_β_1_-integrin affinity: an unexpected mechanism for inducing cell de-adhesion

**DOI:** 10.1186/1471-2172-12-28

**Published:** 2011-05-17

**Authors:** Alexandre Chigaev, Yelena Smagley, Larry A Sklar

**Affiliations:** 1 Department of Pathology and Cancer Center, University of New Mexico Health Sciences Center, Albuquerque, NM 87131, USA

## Abstract

**Background:**

Integrin activation in response to inside-out signaling serves as the basis for rapid leukocyte arrest on endothelium, migration, and mobilization of immune cells. Integrin-dependent adhesion is controlled by the conformational state of the molecule, which is regulated by seven-transmembrane Guanine nucleotide binding Protein-Coupled Receptors (GPCRs). α_4_β_1_-integrin (CD49d/CD29, Very Late Antigen-4, VLA-4) is expressed on leukocytes, hematopoietic progenitors, stem cells, hematopoietic cancer cells, and others. VLA-4 conformation is rapidly up-regulated by inside-out signaling through Gα_i_-coupled GPCRs and down-regulated by Gα_s_-coupled GPCRs. However, other signaling pathways, which include nitric oxide-dependent signaling, have been implicated in the regulation of cell adhesion. The goal of the current report was to study the effect of nitric oxide/cGMP signaling pathway on VLA-4 conformational regulation.

**Results:**

Using fluorescent ligand binding to evaluate the integrin activation state on live cells in real-time, we show that several small molecules, which specifically modulate nitric oxide/cGMP signaling pathway, as well as a cell permeable cGMP analog, can rapidly down-modulate binding of a VLA-4 specific ligand on cells pre-activated through three Gα_i_-coupled receptors: wild type CXCR4, CXCR2 (IL-8RB), and a non-desensitizing mutant of formyl peptide receptor (FPR ΔST). Upon signaling, we detected rapid changes in the ligand dissociation rate. The dissociation rate after inside-out integrin de-activation was similar to the rate for resting cells. In a VLA-4/VCAM-1-specific myeloid cell adhesion system, inhibition of the VLA-4 affinity change by nitric oxide had a statistically significant effect on real-time cell aggregation.

**Conclusions:**

We conclude that nitric oxide/cGMP signaling pathway can rapidly down-modulate the affinity state of the VLA-4 binding pocket, especially under the condition of sustained Gα_i_-coupled GPCR signaling, generated by a non-desensitizing receptor mutant. This suggests a fundamental role of this pathway in de-activation of integrin-dependent cell adhesion.

## Background

Integrins are ubiquitous cell adhesion molecules that play an essential role in the regulation of leukocyte traffic, stem cell mobilization and homing, immune responses, development, hemostasis, and cancer [1-3]. On the cell surface at rest, a variety of integrin exhibit a non-adhesive inactive state and multiple signaling cascades are capable of rapidly and reversibly regulating integrin-dependent cell adhesion. Typically, this regulation is achieved without altering the integrin expression level. Conformational changes within the molecule, together with a spatial reorganization of integrins, are responsible for the rapid modulation of cell adhesion [1,4-6]. Understanding signaling pathways that regulate activation and, especially, inactivation of integrin-mediated cell adhesion is crucial, as integrins are implicated in many human diseases [7-9]. Several existing and emerging drugs for treating inflammatory diseases, anti-angiogenic cancer therapy, anti-thrombotic therapy, and others specifically target integrin molecules [10-12]. Moreover, interfering with integrin activation by targeting "the final steps of activation process" is envisioned as a novel approach for therapeutic intervention in integrin-related pathologies [13].

Very Late Antigen-4, VLA-4, (α_4_β_1_-integrin, CD49d/CD29) is expressed on a majority of peripheral blood leukocytes, hematopoietic progenitors and stem cells, as well as hematopoietic cancer cells [2,14,15]. VLA-4 has the potential to exhibit multiple affinity (conformational) states that mediate tethering, rolling, and firm arrest on VCAM-1 (CD106, Vascular Cell Adhesion Molecule-1) [16-18]. The VLA-4 conformational state is regulated by G protein-coupled receptors (GPCRs) that operate as receptors for multiple chemokines and chemoattractants. The majority of receptors activating VLA-4 are Gα_i_-coupled GPCRs that function by inhibiting adenylate cyclase and inducing calcium mobilization. These include CXCR2, CXCR4, and others [19]. Gα_i_-coupled GPCRs activate integrin by triggering the so-called inside-out signaling pathway [20], which leads to a rapid increase in ligand binding affinity that is translated into the "rapid development of firm adhesion" [18].

Recently, in addition to the inside-out integrin activation pathway, we described a de-activation signaling pathway that can rapidly down-regulate the binding affinity state of the VLA-4 binding pocket. Two Gα_s_-coupled GPCRs (histamine H2 receptor and β2-adrenergic receptors), an adenylyl cyclase activator, and a cell permeable analog of cAMP showed the ability to regulate VLA-4 ligand binding affinity as well as VLA-4/VCAM-1 dependent cell adhesion on live cells in real-time [21]. Based on these findings we hypothesized that other de-activating inside-out signaling pathways might exist. Review of the literature indicated that nitric oxide/cGMP-dependent signaling pathway could be one of them.

Both cAMP/PKA and cGMP/PKG signaling pathways play an inhibitory role in GPCR-induced platelet aggregation and adhesion [22], which is known to be critically dependent on the activation state of platelet integrins [23,24]. Cyclic nucleotide dependent kinases (PKA and PKG) share a strong sequence homology and exhibit overlapping substrate specificity [25]. Nitric oxide signaling is critical for hematopoietic progenitor and stem cell mobilization [26,27], a physiological process that is critically dependent on the interaction between VLA-4 integrin and VCAM-1 [28-32]. Nitric oxide is also shown to antagonize GPCR signaling in muscle cells [33]. The molecular mechanism by which nitric oxide regulates integrin-dependent adhesion is under active investigation. Several reports indicate that direct s-nitrosylation of cytoskeletal proteins [34], or integrins themselves [35], can be involved in the regulation of integrin-dependent adhesion. The goal of the current manuscript was to investigate the effects of exogenous nitric oxide, and other cGMP pathway regulators on VLA-4 conformational regulation on live cells in real-time.

We found that the addition of a nitric oxide donor can rapidly induce dissociation of the VLA-4 specific ligand after cellular activation by any of three GPCRs (CXCR4, CXCR2, and FPR). The effect of nitric oxide was also mimicked by a NO-independent cGMP-cyclase activator, as well as a cell permeable analog of cGMP. This indicates that the integrin deactivation mechanism is intracellular, and suggests that deactivation is not related to direct s-nitrosylation. We also detected rapid changes in the dissociation rate constant (k_off_) of the VLA-4 specific ligand. As shown previously, modulation of the k_off _directly correlates with changes in the VLA-4 ligand binding affinity [14,17]. Finally, using a VLA-4/VCAM-1 specific cell adhesion system, we showed that treatment of cells with a nitric oxide donor diminished GPCR activated cell adhesion to the level of unstimulated (untreated) cells. Taken together, our results indicate that the NO/cGMP signaling pathway can actively down-regulate the affinity of the VLA-4 ligand binding pocket. This observation provides a molecular mechanism for the anti-adhesive activity of nitric oxide donors and drugs that modulate cGMP signaling pathway.

## Results

### Small molecule probes for dissecting the nitric oxide/cGMP pathway

**Figure 1:**
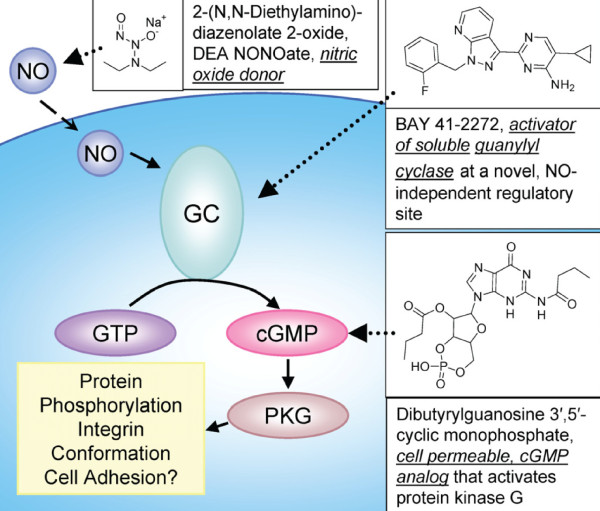
**NO/cGMP signaling cascade and small molecules that modulate this pathway, used in the current study**. Nitric oxide, generated by nitric oxide synthase, diffuses across the plasma membrane and through the cytoplasm. In leukocytes NO reacts with the active site of guanylyl cyclase (guanylate GC), and stimulates the production of the intracellular mediator cyclic GMP (cGMP). Next, cGMP interacts with the cGMP-dependent protein kinase (PKG), which phosphorylates multiple substrates, and participates in signal propagation. Cyclic nucleotide phosphodiesterases (PDEs, not shown) can rapidly hydrolyze cGMP and terminate signal propagation. The NO/cGMP signaling pathway can be specifically targeted using small molecules. The nitric oxide donor provides an exogenous source of NO. The activator of soluble guanylyl cyclase binds to GC, and induces enzyme activation in the absence of NO. The cell permeable analog of cGMP diffuses across the plasma membrane, and thus, activates cGMP-dependent signaling.

The nitric oxide/cGMP signaling pathway has been described in mature leukocytes, platelets, and hematopoietic progenitors. It is composed of soluble guanylyl cyclase (GC) that serves as an intracellular receptor for nitric oxide (Figure 1). Upon binding to NO-sensitive guanylyl cyclase, nitric oxide induces a conformational change resulting in the activation of the enzyme [36], and conversion of GTP to cGMP. Cyclic guanosine monophosphate binding leads to the subsequent activation of the cGMP dependent kinase PKG that phosphorylates multiple substrates, and participates in the regulation of platelet adhesion and aggregation [37].

To study the effects of nitric oxide/cGMP signaling in leukocytes, we selected three small molecules that specifically target this pathway (Figure 1). Diethylamine NONOate can be described as a complex of diethylamine with nitric oxide. It is unstable in aqueous solution and used as nitric oxide donor [38]. BAY 41-2272 is an activator of soluble guanylyl cyclase, which stimulates cGMP production through an NO-independent mechanism [39,40]. N^2^,2'-O-dibutyrylguanosine 3',5'-cyclic monophosphate is a cell permeable cGMP analog that activates protein kinase G [41]. These molecules are shown to stimulate the three initial consecutive steps of the pathway (Figure 1), and therefore, can be used to mimic NO-dependent signaling.

### Nitric oxide donor induces rapid decrease in the binding of VLA-4 specific ligand

**Figure 2:**
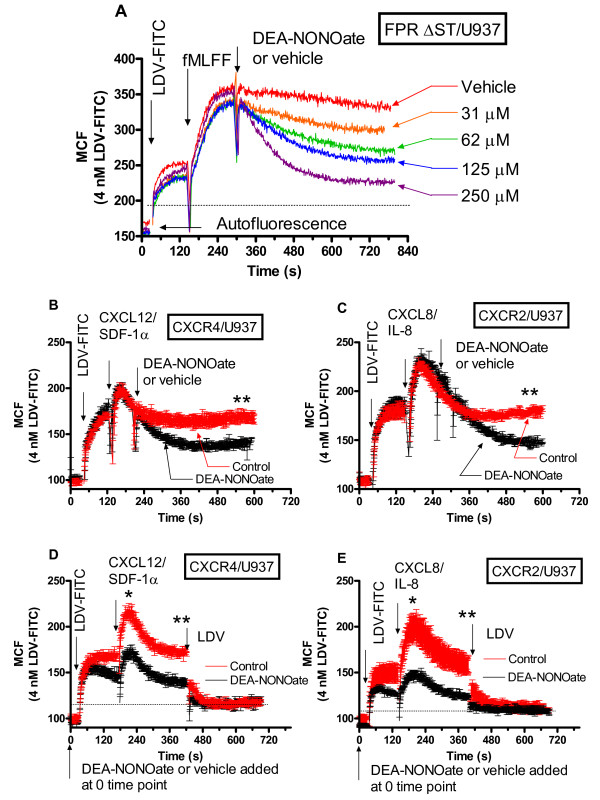
**Effect of nitric oxide addition on binding and dissociation of the LDV-FITC probe on U937 cells, treated with different Gα**_**i**_**-coupled receptor ligands**. LDV-FITC probe binding and dissociation on U937 cells stably transfected with different GPCRs plotted as mean channel fluorescence (MCF) versus time. A, The experiment involved sequential addition of fluorescent LDV-FITC probe (4 nM, below saturation, added 2 min prior to addition of Gαi-coupled receptor ligand, fMLFF, 100 nM), and different concentrations of DEA-NONOate (nitric oxide donor). Dashed line indicates the non-specific binding of the LDV-FITC probe determined using an excess of unlabelled LDV competitor (as shown in Fig. 2D,E). B, The experiment involved sequential addition of fluorescent LDV-FITC probe (4 nM), CXCL12/SDF-1 (12 nM), and DEA-NONOate (250 μM, nitric oxide donor) or vehicle (control). Rapid and reversible binding of the probe reflects the VLA-4 affinity change [14]. C, The experiment involved sequential addition of the fluorescent LDV-FITC probe (4 nM), CXCL8/IL-8 (20 nM), and DEA-NONOate (250 μM, nitric oxide donor) or vehicle (control). D, The experiment involved sequential addition of the DEA-NONOate (250 μM, nitric oxide donor) or vehicle (control) at the 0 time point, and the fluorescent LDV-FITC probe (4 nM), CXCL12/SDF-1 (12 nM). Excess unlabelled competitor LDV (1 μM) is added at the end of the experiment to determine the non-specific binding of the probe (panels D, and E). E, The experiment involved sequential addition of DEA-NONOate (250 μM, nitric oxide donor) or vehicle (control) at the 0 time point, and the fluorescent LDV-FITC probe (4 nM), CXCL8/IL-8 (20 nM) (arrows). According to the unpaired t test, the means are significantly different (p<0.05) at the peak of activation (marked on panels D and E as “*”), and at the steady state (marked on panels B-E as “**”). Experiments shown in the different panels were performed using different instruments, and therefore MCF values are not identical.

Previously, we described and characterized in detail a model ligand an LDV-FITC containing small molecule ([14,42-44], and references therein) for the detection of VLA-4 conformational regulation. This VLA-4 specific fluorescent probe was based on a highly specific α_4_β_1_-integrin inhibitor BIO1211, which contains the Leu-Asp-Val (LDV) ligand binding motif from the alternatively spliced connecting segment-1 (CS-1) peptide of cellular fibronectin [17,45]. We established that integrin affinity changes, detected using this probe, vary in parallel with the natural VLA-4 ligand, human VCAM-1 [46]. For real-time detection of rapid integrin conformational changes, cells were treated with LDV-FITC (Figure 2, first arrow), which was added after establishing a baseline for unstained cells, indicated on Figure 2A as "autofluorescence". Next, data were acquired for 2-3 minutes, and cells were activated with fMLFF (high affinity FPR ligand), CXCL12/SDF-1 (CXCR4 ligand), or CXCL8/IL-8 (CXCR2 ligand), for FPR, CXCR4, CXCR2 transfected cells, respectively (Figure 2A, B, and 2C). The concentration of the LDV-FITC probe used in the experiments (4 nM) was below the dissociation constant (K_d_) for its binding to resting VLA-4 (low affinity state, K_d _~ 12 nM), and above the K_d _for physiologically activated VLA-4 (high affinity state, K_d _~ 1-2 nM) [14]. Therefore, the transition from the low affinity to the high affinity receptor state led to increased binding of the probe (from ~25% to ~70-80% of receptor occupancy, as calculated based on the one site binding equation). The change in occupancy was detected as a rapid increase in the mean channel fluorescence (MCF). This signal increase was sustained for the case of a non-desensitizing mutant of FRP (Figure 2A), and reversible for the wild-type receptors (CXCR4, and CXCR2, Figure 2B,C). Next, cells were treated with the nitric oxide donor, or vehicle (control). Acquisition was re-established, and data were acquired continuously for up to 720-840 s. Addition of the nitric oxide donor resulted in a rapid and dose-dependent decrease in the binding of the VLA-4 specific ligand. In the absence of receptor desensitization, the effect of nitric oxide was more evident in cells transfected with a non-desensitizing mutant of FPR (vehicle, Figure 2A) [47,48]. However, the effect of the nitric oxide donor was statistically significant for both wild-type GPCRs. A faster and more pronounced signal decrease was detected (see black lines in Figure 2B, 2C). To emphasize statistically the difference between control and experimental samples, standard errors of mean are indicated using error bars for every experimental point in Figure 2B, 2C, 2D, 2E.

Next, we studied the effect of nitric oxide donor added prior to cell activation. DEA-NONOate was added at the 0 time point as indicated by the arrow (Figure 2D, 2E). This resulted in a significant decrease in the magnitude of the response for both SDF-1 and IL-8 treated cells. Moreover, the effect of nitric oxide can be detected prior to cell activation. This suggests that at rest a small number of VLA-4 molecules exists in the activated conformation, and addition of nitric oxide donor deactivates these integrins. It worth noting that the nonspecific binding of the LDV-FITC probe remained identical for both control and treated samples (compare sample fluorescence after addition of LDV). Thus, the nitric oxide donor rapidly decreased binding of the VLA-4 specific fluorescent ligand after cell activation through three Gα_i_-coupled GPCRs. Pretreatment with the nitric oxide donor significantly diminished the magnitude of the response.

### Activator of soluble guanylyl cyclase induces a dose-dependent decrease in the binding of the VLA-4 specific ligand

**Figure 3:**
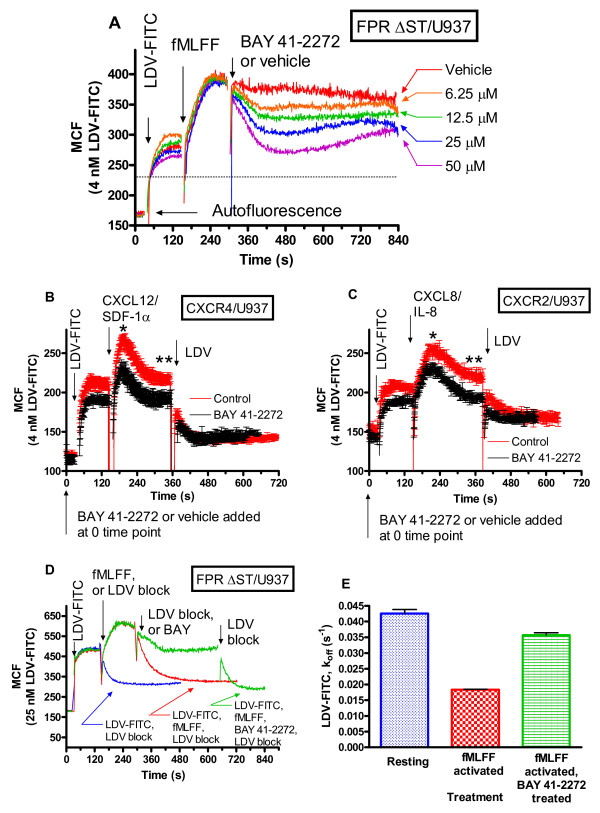
**Effect of guanylyl cyclase activator on binding and dissociation of the LDV-FITC probe on U937 cells, treated with different Gα**_**i**_**-coupled receptor ligands**. LDV-FITC probe binding and dissociation on U937 cells stably transfected with different GPCRs plotted as mean channel fluorescence (MCF) versus time. A, The experiment involved sequential addition of the fluorescent LDV-FITC probe (4 nM, below saturation, added 2 min prior to addition of the Gαi-coupled receptor ligand, fMLFF, 100 nM), and different concentrations of BAY 41-2272 (guanylyl cyclase activator).  B, The experiment involved sequential addition of the BAY 41-2272 (100 μM, guanylyl cyclase activator) or vehicle (control) at the 0 time point, and the fluorescent LDV-FITC probe (4 nM), and CXCL12/SDF-1 (12 nM). C, The experiment involved sequential addition of BAY 41-2272 (100 μM, guanylyl cyclase activator) or vehicle (control) at the 0 time point, and the fluorescent LDV-FITC probe (4 nM), and CXCL8/IL-8 (20 nM). The means are significantly different (p<0.05) at the peak of activation (marked on panels B and C as “*”), and at the steady state (marked in panels B and C as “**”). D, Kinetic analysis of binding and dissociation of the LDV-FITC probe. Cells were sequentially treated with the LDV-FITC probe (25 nM, near saturation), the Gαi-coupled receptor ligand (fMLFF, 100 nM), BAY 41-2272 (guanylyl cyclase activator, 50 μM). At time points indicated by arrows, cells were treated with excess unlabeled LDV containing small molecule (2 μM), and the dissociation of the fluorescent molecule was followed. Dissociation rate constants (koff) were obtained by fitting dissociation curves to a single exponential decay equation. E, Dissociation rate values, obtained in experiments analogous to panel D, summarized as a bar graph showing mean and SEM (n=4). Colors of the dissociation curves in panel D and bars on panel E are matching. The difference between koffs for “resting” and “fMLFF activated”, and between “fMLFF activated” and “fMLFF activated and treated with BAY 41-2272” is statistically significant (P < 0.05) as calculated by one-way ANOVA.

To confirm that the effect of nitric oxide can be mimicked using a nitric oxide-independent activator of soluble guanylyl cyclase, we repeated the experiments shown in Figure 2A using BAY 41-2272 (Figure 3A). Cells, transfected with a non-desensitizing mutant of FPR, were sequentially treated with LDV-FITC (4 nM), fMLFF, vehicle, or indicated concentrations of the soluble guanylyl cyclase activator. We observed a significant decrease in LDV-FITC binding, comparable to the effect induced by the nitric oxide donor (Figure 2A). However, the decrease in LDV-FITC binding was partially reversible. This phenomenon can be rationalized, in terms of the proposed feedback loops that regulate cGMP production. Intracellular cGMP can directly stimulate the catalytic activity of several cyclic nucleotide phosphodiesterases (PDEs) that hydrolyze cGMP [49-51]. Another possibility is activation of PDEs through phosphorylation by cGMP-dependent protein kinase (PKG) (Figure 1) [50-53].

Next, we studied the effect of the nitric oxide-independent activator of soluble guanylyl cyclase added prior to cell activation. BAY 41-2272 was added at the 0 time point as indicated by the arrow (Figure 3B, 3C). This resulted in a decrease in the magnitude of the response for both SDF-1 and IL-8 treated cells in a manner comparable to the effect of nitric oxide donor. Similarly, the effect of activator of soluble guanylyl cyclase can be detected prior to cell activation. Thus, the nitric oxide-independent activator of soluble guanylyl cyclase induces a dose-dependent decrease in binding of the VLA-4 specific ligand, and pretreatment with the activator of soluble guanylyl cyclase significantly diminished the magnitude of the response after activation.

### Dissociation rate analysis revealed rapid changes in the dissociation rate of the VLA-4 specific ligand

As shown previously, for different states of VLA-4 affinity, the LDV-FITC equilibrium dissociation constant K_d _varied inversely with the dissociation rate constant (k_off_). This implies that the ligand association rate constant is essentially independent of receptor conformation (for example see Table I in [17]), or Table I in [14]). Therefore, the dissociation rate analysis can be used to assess the affinity state of the VLA-4 integrin binding pocket.

To saturate the majority of low affinity sites, cells transfected with a non-desensitizing mutant of FPR were preincubated with a higher concentration of the VLA-4 specific ligand (25 nM). Since the K_d _for the low affinity state is ~12 nM (Table I in [14]), at 25 nM ~70% of sites are occupied before activation. Next, an excess of the unlabeled LDV competitor (labeled on Figure 3D as "LDV block") is added to induce dissociation of the LDV-FITC probe. After activation by fMLFF, because of the rapid affinity change, little additional binding of the probe was seen (Figure 3D, green and red lines). Addition of the nitric oxide-independent activator of the soluble guanylyl cyclase returned the binding of the probe to a level similar to the binding before fMLFF addition.

Next, the regions of the ligand-binding curves corresponding to the dissociation of the LDV-FITC probe were fitted to a single exponential decay equation. The resulting dissociation rate constants (k_off_, s^-1^) are shown graphically in Figure 3E. At rest, the majority of the VLA-4 molecules exhibit rapid probe dissociation, corresponding to the low affinity state of the ligand binding pocket (Figure 3D, 3E, blue curve "LDV-FITC, LDV block", k_off _~ 0.04 ± 0.001 s^-1^). After cell activation by fMLFF, the dissociation rate was significantly slower (Figure 3D, 3E, red curve "LDV-FITC, fMLFF, LDV block", k_off _~ 0.018 ± 0.0001 s^-1^). The slower k_off _corresponds to higher ligand binding affinity [14,17,46]. After the addition of the nitric oxide-independent activator of soluble guanylyl cyclase, dissociation rates were comparable to the rate for the resting state (Figure 3D, 3E, green curve "LDV-FITC, fMLFF, BAY 41-2272, LDV block", k_off _~ 0.036 ± 0.0007 s^-1^). This suggests that activation of guanylyl cyclase can actively down-regulate the affinity state of the VLA-4 integrin ligand binding pocket, even under the condition with the continuously signaling non-desensitizing GPCR mutant. The affinity state induced by guanylyl cyclase activator was quantitatively similar to the resting state before activation. The resting VLA-4 conformation on U937 cells exhibits the lowest physiological affinity. It is worth noting, that this result is comparable to the effect of Gα_s_-coupled GPCRs on VLA-4 conformation (compare Figure 3D in the current manuscript and Figure 2C, 2D in [21]). This result is especially interesting in light of the structural relationship of the two second messengers cAMP and cGMP, originating from these signaling pathways.

### Dibutyrylguanosine 3',5'-cyclic monophosphate induces rapid and reversible changes in the binding of the VLA-4 specific ligand

**Figure 4:**
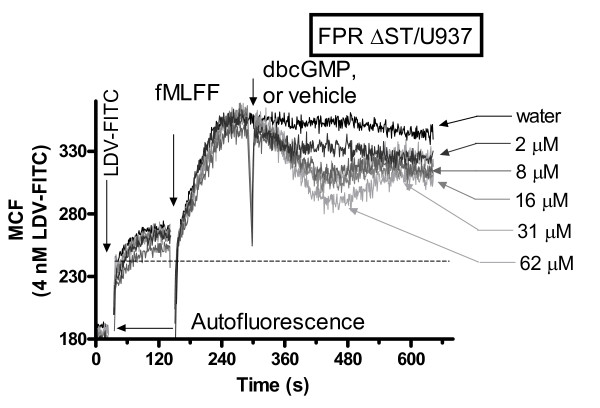
**Effect of the cell permeable analog of cGMP on binding and dissociation of the LDV-FITC probe on U937 cells stably transfected with the non-desensitizing mutant of FPR**. LDV-FITC probe binding and dissociation on U937 cells stably transfected with the non-desensitizing mutant of FPR plotted as mean channel fluorescence (MCF) versus time. The experiment involved sequential addition of the fluorescent LDV-FITC probe (4 nM, below saturation, added 2 min prior to addition of the Gα_i_-coupled receptor ligand, fMLFF, 100 nM), and different concentrations of dibutyrylguanosine 3',5'-cyclic monophosphate (cell permeable cGMP analog) (arrows). Control cells were treated with vehicle. The MCF value corresponding to cell autofluorescence is indicated by the horizontal arrow. Dashed line indicates the non-specific binding of the LDV-FITC probe determined using excess unlabelled LDV competitor (as shown on Figure 3B). Rapid and reversible binding of the probe reflects the VLA-4 affinity change [14]. Curves are means out of two independent determinations calculated on a point-by-point basis (n = 2).

Next, we studied the effect of the cell permeable analog of cGMP on real-time binding of the LDV-FITC probe (Figure 4). Addition of dbcGMP induced a dose-dependent decrease in the binding of the probe. However, the effect of dbcGMP was reversible. These kinetics are compatible with negative feedback loops that regulate cGMP dependent signaling. Activation of PDEs directly by cGMP binding, or indirectly after being phosphorylated by a cGMP dependent kinase (PKG), has been previously reported [49-53].

Thus, all three probes specifically targeting the NO/cGMP pathway (the nitric oxide donor, the nitric oxide-independent activator of soluble guanylyl cyclase, and the cell permeable analog of cGMP) were found to decrease binding of the VLA-4 specific ligand, with similar kinetics, after cell activation through Gα_i_-coupled GPCRs. To study the effects of NO/cGMP signaling on cell aggregation, we used a model system, consisting of U937 cells, stably transfected with GPCR in the experiments described above (Figures 2, 3, 4), and a mouse melanoma cell line stably transfected with human VCAM-1. The unlabelled VLA-4 specific ligand (LDV), analogous to the LDV-FITC probe, was used to identify VLA-4/VCAM-1 specific cell aggregation. This model system has been described and characterized previously [42,46,54,55].

### The effect of nitric oxide/cGMP signaling pathway activation on VLA-4 -VCAM-1 dependent cell adhesion

**Figure 5:**
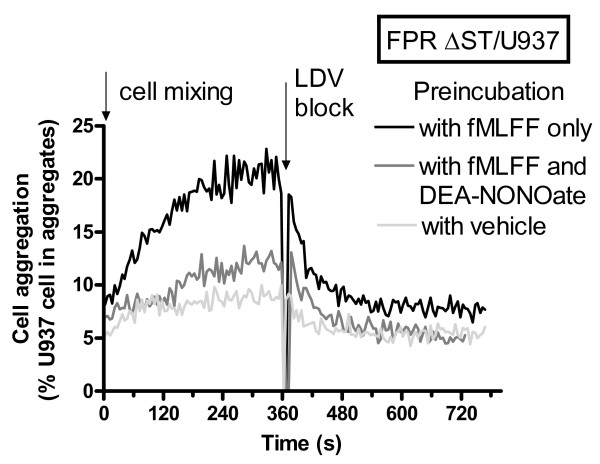
**Changes in cell adhesion between U937 FPR (ΔST) and VCAM-1-transfected B78H1 cells in the resting state and in response to receptor stimulation**. Real-time aggregation experiments were conducted as described under "Methods". U937/ΔST FPR stably transfected cells, which constitutively express VLA-4, were labeled with red fluorescent dye, and B78H1/VCAM-1 transfectants were stained with green fluorescent dye. Labeled cells were preincubated for 10 min at 37°C with fMLFF only (100 nM, activated control), DMSO (vehicle, resting cells control), or with fMLFF and DEA-NONOate (250 μM, nitric oxide donor) in a manner analogous to the experiment showed in Figure 2A. Next, cells were mixed and real-time cell aggregation (red and green double positive events) was followed. To determine the level of VLA-4 dependent cell aggregation, 6 min after cell mixing, excess unlabelled VLA-4 specific ligand was added (arrow, LDV block, 2 μM). This induced rapid cellular disaggregation to the level of non-specific binding. A representative experiment out of three experiments is shown.

Prior to the experiment, individual cell populations were stained with either of two fluorescent dyes (red and green). Next, the cell populations were mixed, and the appearance of double positive events, representing cellular aggregates, was followed in real-time by flow cytometry (see Figure 1, 2, 3 in [55] for method details). Because nitric oxide represents a "natural" signaling molecule, and the effect of nitric oxide was not reversible during the first several hundred seconds after treatment (Figure 2A), for aggregation experiments cell were treated with the NO-donor (Figure 5). Resting (unstimulated) cells showed a very small increase in the % U937 cells in the cell aggregate (Figure 5, light gray line, labeled "with vehicle"). Inside-out activation resulted in a rapid increase in cell aggregation during the first six minutes after mixing the cell populations (Figure 5, black line, labeled "with fMLFF only"). Addition of the unlabelled VLA-4 specific ligand "LDV block" resulted in rapid cellular disaggregation, indicating that the majority of aggregates were VLA-4 dependent. The overall extent of activated cell aggregation was similar to previously published data [46]. Pretreatment of U937 cells with fMLFF, and subsequently with nitric oxide donor, in a manner similar to the Figure 2A, abolished fMLFF-dependent cellular aggregation (gray line, labeled "with fMLFF and DEA-NONOate"). In fact, cell aggregation in this experiment was very similar to the aggregation of the resting cell (untreated control). Thus, treatment of activated cells with NO-donor only abolished the effect of GPCR-dependent cell activation, and did not affect resting cell aggregation. This result is additionally supported by the LDV-FITC ligand binding kinetics data (Figure 3B, 3C). Activation of guanylyl cyclase induced a rapid decrease of the VLA-4 ligand binding affinity to a level that was quantitatively similar to the resting state. Thus, the NO/cGMP signaling pathway provides an antagonistic signal that can rapidly and actively decrease the affinity state of the VLA-4 ligand binding pocket, and this results in the modulation of VLA-4/VCAM-1 dependent cellular aggregation.

## Discussion

### Inside-out deactivation of integrins

A current paradigm of the inside-out activation of integrins implies an instantaneous triggering of integrin conformational changes, where a chemokine signal appears to be closely apposed to the integrin [56]. An "updated" adhesion cascade includes several steps in addition to the traditional tethering, rolling, and arrest [57]. While integrin adhesion research is largely focused on activating pathways, the inhibitory Gα_s_-coupled GPCR/cAMP-dependent signaling pathways is acknowledged for platelet regulation [58]. The relative lack of interest in the integrin deactivation pathways is potentially compensated by the identification of antagonists that competitively block adhesive interactions, and thus, provide a desirable therapeutic effect [59].

However, it is arguable that deactivation of the signaling pathway is as appealing as a direct blockade of the activating signaling using receptor antagonists. It was established, that in order to induce a half-optimal elevation of the signal in leukocytes, only a very small fraction of occupied cellular receptors is required. In some cases, this fraction may be less than 0.1% of the total number of receptors [60]. This is dependent on significant signal amplification for both stimulatory and inhibitory pathways [61]. Therefore, from a therapeutic point of view, it would be very difficult to completely block the occupancy of activating chemokine receptors using receptor-specific antagonists. A small fraction of activating receptors occupied by the ligand, may be sufficient to trigger the adhesion signal. A plausible scenario would be to take advantage of natural regulatory pathways to counteract unwanted signaling, especially because antagonistic pathways potentially have similar amplification capacity [60,61].

### NO-dependent VLA-4 deactivation and hematopoietic stem cell mobilization

The VLA-4 integrin is critical for the interaction of hematopoietic progenitors and stromal cells [2,3]. Blocking of the VLA-4/VCAM-1 interaction using anti-VLA-4 antibodies, small molecule competitive as well as allosteric VLA-4 antagonists, results in the mobilization of progenitors into the peripheral blood [28-32,62]. Endothelial nitric oxide synthase (eNOS), one of the major enzymes, producing nitric oxide in the vasculature, is essential for the mobilization of stem and progenitor cells from the bone marrow stem cell niche. Mice lacking eNOS showed a defect in progenitor mobilization [26]. Nitric oxide synthase-derived nitric oxide regulates the bone marrow environment, and is envisioned as a direct mediator of cell mobilization [27]. Our current finding that nitric oxide/cGMP signaling pathway can actively down-regulate VLA-4 affinity, even under conditions of constant signaling, induced by a non-desensitizing mutant of GPCR, indicates that VLA-4 conformational deactivation provides a plausible explanation for the molecular basics of nitric oxide signaling-induced progenitor mobilization.

### Future directions

Several reports indicate that other signaling pathways actively down-regulate integrin affinity and cell adhesion. For example, cyclic cytidine 3',5'-monophosphate (cCMP) was shown to modulate leukocyte activation [63], to inhibit platelet activation, and to signal through cGMP-dependent protein kinase (PKG) [64]. Although, the existence and metabolism of cCMP in mammals is uncertain [65], recent report suggests that the adenylyl cyclase toxin edema factor and adenylate cyclase-hemolysin (CyaA), produced by *Bacillus anthracis *and *Bordetella pertussis*, can catalyze the formation of cCMP, and other cyclic nucleotides [66]. The ability of cCMP to down-modulate immune cell adhesion, and thus, decrease immune response could be a part of a host immunity subversion strategy. Nonetheless the search for novel inside-out deactivating pathways is justified, especially, if we would like to understand and take full advantage of natural regulatory mechanisms that evolved to enable anti-adhesive signaling.

Do deactivating signaling pathways operate on other integrins? A large body of the literature suggests that major integrins, expressed on blood cells and platelets, behave in a similar way. Integrin-dependent platelet aggregation and adhesion are known to be actively regulated by cyclic nucleotides [67]. Cyclic AMP is known to rapidly decrease LFA-1 dependent cell adhesion [68], and binding of the ligand to activated LFA-1 can be rapidly and reversible down-modulated in a manner identical to VLA-4 [21,69]. So far the information is limited for other integrins.

Are there existing drugs in use today that take advantage of these signaling pathways? The success of PDE5 inhibitors, blocking hydrolysis of cGMP, and currently used for the treatment of erectile dysfunction stimulated significant interest in other therapeutic applications of the NO/cGMP-dependent signaling pathway. Studies on different PDE inhibitors show potential in anti-inflammatory therapy. Currently, a large number of preclinical *in vivo *studies on PDE inhibitors exhibit decreased cell recruitment, activation of inflammatory cells and physiological changes in lung function in asthma, chronic obstructive pulmonary disease, and others [70,71]. cAMP phosphodiesterase-4 inhibitor showed anti-inflammatory activity in vitro and in a model of psoriasis [72]. Thus, our finding that NO/cGMP pathway directly regulates integrin-dependent immune cell adhesion provides the rationale for repositioning of existing drugs toward pathologies, where integrin-mediated excessive immune cell adhesion/recruitment is envisioned to be detrimental.

## Conclusions

We conclude that the nitric oxide/cGMP signalling pathway dramatically decreases the up-regulation of VLA-4 integrin ligand-binding affinity, when triggered prior to inside-out integrin activation, and rapidly down-modulates VLA-4 affinity, when induced after integrin activation. This conformational change results in a significant down-regulation of VLA-4-dependent cell adhesion, suggesting a major role of this pathway in the regulation of inside-out integrin de-activation and cell de-adhesion (mobilization).

## Methods

### Materials

The VLA-4 specific ligand [14,46,47] 4-((N'-2-methylphenyl)ureido)-phenylacetyl-L-leucyl-L-aspartyl-L-valyl-L-prolyl-L-alanyl-L-alanyl-L-lysine (LDV containing small molecule), and its FITC-conjugated analog (LDV-FITC) were synthesized at Commonwealth Biotechnologies. Human recombinant CXCL12/SDF-1α, and recombinant human CXCL8/IL-8 were from R&D Systems. All other reagents were from Sigma-Aldrich. Stock solutions were prepared in DMSO, at concentrations ~1000 fold higher than the final concentration. Usually, 1 μl of stock solution was added to 1 ml of cell suspension yielding a final DMSO concentration of 0.1%. Control samples were treated with an equal amount of pure DMSO (vehicle). CXCL12/SDF-1α and CXCL8/IL-8 solutions were prepared using water, and used according to manufacturer's instructions.

### Cell Lines and Transfectant Construct

The human histiocytic lymphoma cell line U937 and mouse melanoma cell line B78H1 were purchased from ATCC. Wild type CXCR4 (CD184) receptor, and CXCR2, IL-8RB, (CD128b, CD182) stably transfected U937 cells, and site-directed mutants of the FPR (non-desensitizing mutant of FPR ΔST) in U937 cells were prepared as described [73] and were a gift of Dr. Eric Prossnitz (University of New Mexico). For transfection of B78H1 cells, full-length human VCAM-1 cDNA was a kind gift from Dr. Roy Lobb of Biogen Inc. The original construct [74] was subcloned into the pTRACER vector (Invitrogen). Transfection into B78H1 was done using the LipofectAMINE Reagent (Invitrogen). High expressors were selected using the MoFlo Flow Cytometer (DakoCytomation). Cells were grown in RPMI 1640 (supplemented with 2 mm l-glutamine, 100 units/ml penicillin, 100 g/ml streptomycin, 10 mm HEPES, pH 7.4, and 10% heat-inactivated fetal bovine serum) and then harvested and resuspended in 1 ml of HEPES buffer (110 mM NaCl, 10 mM KCl, 10 mM glucose, 1 mM MgCl_2_, 1.5 mM CaCl_2_, and 30 mm HEPES, pH 7.4) containing 0.1% human serum albumin and stored on ice. The buffer was depleted of lipopolysaccharide by affinity chromatography over polymyxin B sepharose (Detoxigel; Pierce Scientific). Cells were counted using the Coulter Multisizer/Z2 analyzer (Beckman Coulter). For experiments, cells were suspended in the same HEPES buffer at 1 × 10^6 ^cells/ml and warmed to 37°C. Alternatively, cells were resuspended in warm RPMI (37°C) and used immediately.

### Kinetic Analysis of Binding and Dissociation of VLA-4 Specific Ligand

Kinetic analysis of the binding and dissociation of the LDV-FITC probe was described previously [14,46]. Briefly, cells (1 × 10^6 ^cells/ml) were preincubated in HEPES buffer containing 0.1% HSA or RPMI under different incubating conditions for 10-20 min at 37°C. Flow cytometric data were acquired for up to 1024 s at 37°C while the samples were stirred continuously at 300 rpm with a 5 × 2 mm magnetic stir bar (Bel-Art Products). For real-time affinity activation experiments, 4 nM LDV-FITC was added after establishing a baseline for unstained cells marked on figures as "autofluorescence". Next, different ligands were added and acquisition was re-established, creating a 5-10 s gap in the time course. For activation, cells were treated with different GPCR ligands at saturating concentration (10 times or higher than K_d_). In several experiments cells were treated sequentially with two different compounds. Acquisition was re-established, and data were acquired continuously for up to 1024 s. The concentration of the LDV-FITC probe used in the experiments (4 nM) was below the dissociation constant (K_d_) for its binding to resting VLA-4 (low affinity state, K_d _~ 12 nM), and above the K_d _for physiologically activated VLA-4 (high affinity state, K_d _~ 1-2 nM) [14]. Therefore, the transition from the low affinity to the high affinity receptor state led to increased binding of the probe (from ~25% to ~70-80% of receptor occupancy, as calculated based on the one site binding equation), which was detected as an increase in the mean channel fluorescence (MCF). For kinetic dissociation measurements, cell samples were preincubated with the fluorescent probe (25 nM), treated with excess unlabeled LDV containing small molecule (2 μM) and the dissociation of the fluorescent molecule was followed. The resulting data were converted to MCF *versus *time using FCSQuery software developed by Dr. Bruce Edwards (University of New Mexico).

### Cell Adhesion Assay

The cell suspension adhesion assay has been described previously [46,55]. Briefly, U937/ΔST FPR stably transfected cells were labeled with red fluorescent PKH26GL dye, and B78H1/VCAM-1 transfectants were stained with green fluorescent PKH67GL dye (Sigma-Aldrich). Labeled cells were washed, resuspended in HEPES buffer supplemented with 0.1% HSA and stored on ice until used in assays. Control U937 cells were preincubated with the 1 μM LDV-containing small molecule for blocking adhesion. Prior to data acquisition, cells were warmed to 37°C for 10 min separately and then mixed. During data acquisition, the samples were stirred with a 5 × 2-mm magnetic stir bar (Bel-Art Products, Pequannock, NJ) at 300 rpm and kept at 37°C. For stimulation, cells were treated with appropriate GPCR ligands at saturating concentration (10 times or higher than K_d_). In several experiments cells were treated sequentially with two different compounds. The number of cell aggregates containing U937 adherent to B78H1/VCAM-1 (red and green cofluorescent particles) as well as the number of singlets (red or green fluorescent particles, FL2 and FL1 in FACScan flow cytometer) were followed in real-time. The percentage of aggregates was calculated as follows: % U937 cells in aggregates = number of aggregates/(number of aggregates + number of U937 singlets)) × 100. Experiments were done using a FACScan flow cytometer and Cell Quest software (Becton Dickinson, San Jose, CA). The data were converted to number of singlets/aggregates *versus *time using FCSQuery software developed by Dr. Bruce Edwards (University of New Mexico).

### Statistical analysis

Curve fits and statistics were performed using GraphPad Prism (GraphPad Prism version 4.00 for Windows, GraphPad Software, San Diego, CA). Each experiment was repeated at least three times. The experimental curves represent the mean of two or more independent runs. SEM was calculated using GraphPad Prism. To estimate the statistical significance of the difference between control and treated samples (as Figures. 2B, 2C, 2D, 2E, and 3B, 3C), the sections of the kinetic curves at the peak of activation and after the steady state was reached (total of 30-80 seconds indicated on Figs. using "*" for the peak and "**" for the steady state) were compared using the unpaired t test (GraphPad Prism version 4.00 for Windows, GraphPad Software, San Diego, CA).

## Abbreviations

cAMP: (adenosine 3',5'-cyclophosphate); BAY 41-2272: (3-(4-Amino-5-cyclopropylpyrimidin-2-yl)-1-(2-fluorobenzyl)-1H-pyrazolo[3,4-b]pyridine, activator of soluble guanylate cyclase); DEA-NONOate: (2-(N,N-Diethylamino)-diazenolate, nitric oxide donor); cGMP: (guanosine 3',5'-cyclic monophosphate); dbcGMP: (N^2^,2'-O-Dibutyrylguanosine 3',5'-cyclic monophosphate); fMLFF: (N-formyl-L-methionyl-L-leucyl-L-phenylalanyl-L-phenylalanine, formyl peptide); FPR: (formyl peptide receptor 1); GC: (guanylate cyclase, guanylyl cyclase); GPCR: (guanine nucleotide binding protein coupled receptor); HAS: (human serum albumin); HEPES: (4-(2-hydroxyethyl)-1-piperazineethanesulfonic acid); IL-8/CXCL8: (Interleukin-8); LDV: containing small molecule (4-((N'-2-methylphenyl)ureido)-phenylacetyl-L-leucyl-L-aspartyl-L-valyl-L-prolyl-L-alanyl-L-alanyl-L-lysine); LDV-FITC: containing small molecule (4-((N'-2-methylphenyl)ureido)-phenylacetyl-L-leucyl-L-aspartyl-L-valyl-L-prolyl-L-alanyl-L-alanyl-L-lysine-FITC); MCF: (mean channel fluorescence, equivalent of mean fluorescence intensity); PKG: (cGMP-dependent protein kinase); SDF-1: (stromal cell-derived factor-1, CXCL12); VCAM-1: (vascular cell adhesion molecule 1, CD106); VLA-4: (very late antigen 4, CD49d/CD29, α_4_β_1 _integrin).

## Authors' contributions

AC designed the study, carried out ligand binding experiments, and wrote the manuscript. YS carried out ligand binding and aggregation experiments. LAS participated in study coordination. All authors read and approved the final manuscript.
